# Stock price dynamics prediction based on multi-scale fractals and deep learning

**DOI:** 10.1371/journal.pone.0335554

**Published:** 2025-12-02

**Authors:** Yuanyuan Du, Ye Tian

**Affiliations:** 1 Henan Polytechnic Institute, School of Economics and Trade, Nanyang, Henan, China; 2 Henan Polytechnic Institute, School of Economics and Trade, Nanyang, Henan, China; Chosun University, KOREA, REPUBLIC OF

## Abstract

The complexity of stock price fluctuations stems from its multi-scale characteristics, nonlinear dynamic characteristics, and fractal structure. To better capture the fractal characteristics of stock prices, this paper creatively proposes a prediction method based on fractal feature extraction and deep learning. First, the generalized Hurst exponent *Hq*, high-order fractal dimension *FDq*, and multifractal spectrum *MFSq* are combined to characterize the long-range correlation and local complexity of stock price series from different scales. In addition, *Rényi* entropy and generalized fractional Brownian motion (GFBM) are introduced to enhance the descriptive ability of features. Secondly, a multi-scale fractal feature fusion mechanism (MSA) is designed to achieve feature aggregation in the time-frequency domain to improve the adaptability of the model to the nonlinear fluctuation pattern of stock prices. Finally, a multi-scale fractal loss function is constructed to integrate *Rényi* error, *Hölder* constraint and fractal spectrum deviation to enhance the ability of the model to maintain fractal structure. Experimental results show that the prediction performance of this method on multiple real market data is better than that of existing methods, and it shows better performance in terms of accuracy, stability and extreme volatility processing ability. This study provides a new theoretical framework for financial time series analysis and a new idea for the application of fractal theory in financial forecasting.

## 1. Introduction

Stock price fluctuation prediction is an important topic in financial market research and has a wide range of applications in asset pricing, investment analysis, risk management [1–3]. However, the stock market is affected by a variety of complex factors and presents nonlinear, non-stationary, and multi-scale fluctuation characteristics. This makes traditional models and conventional machine learning methods have certain limitations in describing stock price trends [4]. Fractal theory provides an effective tool for studying the complex dynamics of the financial market, which can describe the correlation and local structural characteristics of stock price fluctuations [5]. At the same time, deep learning performs well in processing nonlinear data patterns, can autonomously extract complex features, and improve prediction accuracy. Therefore, combining fractal features with deep learning can help to more comprehensively analyze the law of stock price fluctuations and provide more accurate model support for market trend prediction [6,7]. Below we briefly introduce the application of fractal theory in neural networks and financial economics.

Fractional calculus has made significant progress in the field of neural networks, and the theory can discover more detailed data features [8,9]. Existing methods mainly introduce fractal theory to update and iterate network parameters in real time. Parameter update in neural networks is trained by back propagation algorithm, and the key to back propagation is gradient descent method. Gradient descent method is an optimization algorithm that finds the minimum value of loss function by updating network parameters. [10] proposed a regularized fractional-order deep back-propagation neural network model, and optimized the proposed network using the fractional-order gradient descent method with Caputo derivatives, effectively avoiding overfitting. [11] proposed a fractional-order gradient descent method for neural network back-propagation training, but the fractional-order chain rule given was very complex and not convenient for large-scale optimization calculations. [12] proposed a new scheme based on physical information neural network (PINN) to solve fractional-order differential equations (FDEs) based on Caputo derivatives. Through trial solutions based on functional connection theory, approximate solutions for single FDEs and FDE systems were obtained. In the study of ocular tumor diagnosis, [13] combined the Caputo fractional-order gradient descent method (CFGD) with the cuckoo search algorithm (CSA) to improve the accuracy and convergence speed. This study demonstrated the potential of deep learning technology and the proposed optimizer in accurately identifying ocular tumors.

To improve the accuracy of stock price fluctuation prediction, [14] proposed a new multi-heterogeneous self-paced ensemble learning framework. The model uses pairwise comparison of multi-period data and combines the maximum correlation and minimum redundancy methods to select the optimal feature subset, effectively solving the high-dimensional problem. [15] proposed a carbon trading market financial risk prediction auxiliary decision-making model based on a deep neural network. By selecting four perspectives of energy price, climate environment, carbon market price and macroeconomics as input, the influencing factors of carbon emission rights price were analyzed. In the big data environment, [16] developed a feature selection based on adversarial antlion optimizer. For big data management in the financial field, a new prediction algorithm was designed to select the optimal feature subset, which helps to improve the classification results. In response to the problem of large stock price fluctuations, [17] captured small fluctuations by incorporating chaos theory. The model focuses on three key economic variables: interest rate, investment demand and price index, capturing their interactions in chaotic financial systems. In numerical simulations, the author combines chaos theory with financial market analysis, providing valuable insights for understanding economic fluctuations and complex market behaviors.

Although fractional differentials have achieved great success in solving stock price prediction, existing methods still have certain shortcomings when dealing with the complex dynamic characteristics of the market. On the one hand, although the existing deep learning models have powerful feature extraction capabilities, they still have limitations in capturing the fractal structure of stock price series. On the other hand, the current feature fusion strategy lacks targeted modeling of fractal features when fusing multi-scale information, which affects the prediction stability of the model. In addition, common loss functions are usually based on mean square error or cross entropy, which fails to fully consider the fractal feature constraints of stock price fluctuations, resulting in insufficient robustness of the model under extreme market conditions. In response to the above problems, this paper proposes a stock price prediction method that combines fractals and deep learning. The main contributions of this paper are as follows:

- A fractal-based feature extraction method is proposed for the nonlinear and multi-scale fluctuation characteristics of stock prices. The fractal features of stock prices are extracted by combining the generalized Hurst index H_q, the higher-order fractal dimension FD_q and the multi-fractal spectrum MFS_q to more accurately characterize the long-term correlation and local complexity of stock prices.
- In order to optimize feature fusion, a multi-scale fractal feature fusion mechanism is proposed. Combined with the fractal attention mechanism and non-integer order wavelet transform, feature aggregation is performed in the time-frequency domain to enhance the model’s perception of fluctuation patterns at different scales.
- In order to improve the robustness of prediction and the ability to maintain fractal structure, a multi-scale fractal loss function (MSFL) is designed to integrate Rényi error, Hölder constraint and fractal spectrum deviation. While optimizing the traditional error metric, fractal structure constraints are introduced to improve the adaptability and stability of the model under extreme market conditions.

The rest of this paper is arranged as follows: The second part is the methodology. The third part is the experimental comparison. Finally, there are conclusions and prospects.

## 2. Methodology

**Fig 1:**
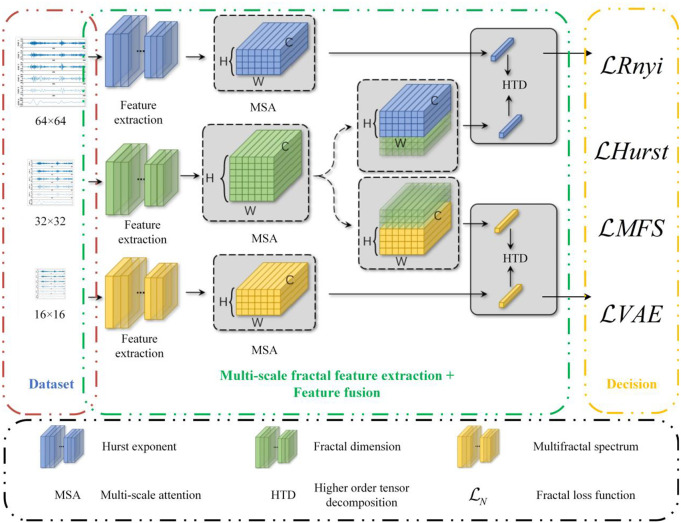
The overall framework of our proposed stock price dynamics prediction method.

This study constructs a stock price fluctuation prediction framework that integrates fractal features and deep learning to fully capture the multi-scale dynamic characteristics of the market and improve prediction accuracy [5,18]. The framework mainly includes three core modules: fractal feature extraction, attention feature fusion, and prediction layer, as shown in Fig 1. First, in the fractal feature extraction stage, three feature channels, namely the Hurst index, fractal dimension, and multi-fractal spectrum, are constructed from the stock price time series to measure the long memory, complexity, and multi-scale inhomogeneity of the market, respectively, providing rich feature input for deep learning. Subsequently, the extracted fractal features are adaptively fused using a multi-channel attention mechanism. Through channel weighting, temporal attention, and feature interaction, the key information expression is enhanced and the redundant effect is reduced, thereby improving the feature learning ability of the model. Finally, in the prediction layer, the stock price fluctuation modeling is carried out by combining a deep neural network, and the fractal loss function is introduced. Through optimization strategies such as generalized *Rényi* entropy, *Hurst* constraint terms, and non-integer order error terms, the model is ensured to fully retain the fractal structure of stock price fluctuations during the learning process, thereby improving the prediction stability and generalization ability. This framework not only strengthens the modeling capabilities of market fractal characteristics, but also effectively combines the time series pattern mining capabilities of deep learning, providing a more robust and interpretable solution for stock price fluctuation prediction. Below we introduce each module in detail.

### 2.1. Fractal feature extraction

In the prediction of stock price fluctuations, traditional time series analysis methods are difficult to effectively capture the multi-scale self-similarity and complex nonlinear fluctuation characteristics of stock prices [19]. Therefore, we use a method based on high-order fractal features to extract the fractal characteristics of stock prices from multiple perspectives such as Hurst index, fractal dimension, and multifractal spectrum. In addition, we combine generalized fractional Brownian motion (GFBM), generalized *Rényi* entropy, and generalized fractional Fourier transform (GFRFT) tools to enrich the fractal theory framework and enhance the feature expression ability of the model [20].

#### 2.1.1. High-order calculation method of Hurst index.

Hurst index *H* is an important indicator to measure the long-term dependence of time series, usually calculated by *R/S* analysis or *DFA* method [21]. In order to improve the ability to characterize stock price fluctuations, we introduce the Hurst index modified by generalized *Rényi* entropy and high-order *R/S* analysis.The steps of calculating Hurst index by traditional R/S analysis are as follows:

Step 1: Let the stock price time series be *X(t)*, and calculate the mean of the subintervals of length *n*:


$$\bar{X}n=\frac{\text{1}}{n}\sum t=1^{n}X(t)$$ (1)

Step 2: Calculate the cumulative deviation:


$$Y(t)=\sum_{i=1}^{t}(X(i)-{\bar{X}}_{n})$$ (2)

Step 3. Calculate the range *R(n)* and standard deviation *S(n)*:


$$R(n)=\max(Y(t))-\min(Y(t)),S(n)=\sqrt{\frac{\text{1}}{n}\sum_{t=1}^{n}(X(t)-{\bar{X}}_{n}{)}^{2}}$$ (3)

Step 4.: Calculate the *R/S* statistic and fit a power law relationship:


$$\begin{array}{c} E\left[\frac{R(n)}{S(n)}\right]\sim n^{H} \end{array}$$ (4)

Among them, H is obtained by regressing log(R(n)/S(n)) and logn.

In order to more comprehensively measure the fractal characteristics of stock prices at different scales, we introduce the generalized *Rényi* entropy correction:


$$H_{q}^{\left(\alpha ,\beta \right)}=\underset{n\to \infty }{\lim}\frac{\log\sum_{i=1}^{N}P_{i}^{\alpha }{\left|X(i+n)-X(i)\right|}^{\beta }}{q\log n}$$ (5)

Among them, $P_{i}=\frac{|X(i){|}^{\alpha }}{\sum_{j}|X(j){|}^{\alpha }}$ is the fractal measure; (α,β) is the control weight preference; when α=0,β=2, it degenerates into the traditional *Hurst* index calculation method.

#### 2.1.2. High-order calculation method of fractal dimension.

Fractal dimension (FD) reflects the complexity and self-similarity of time series [22]. We use fractional box counting method and generalized *Kolmogorov-Smirnov* statistic (GKS) for correction. The traditional fractal dimension calculation method is as follows:


$$FD=\underset{\int \to 0}{\lim}\frac{\log N(\int )}{\log(1/\int )}$$ (6)

Where N(∫) is the minimum number of boxes required to cover the time series when the scale is ∫. In order to more carefully characterize the multi-scale characteristics of stock price data, we use a non-integer order covering method:


$$FD_{q}=\underset{\int \to 0}{\lim}\frac{\log\sum_{i}{\left(\frac{N_{i}(\int )}{N_{total}(\int )}\right)}^{q}}{\left(q-1)\log(1/\int \right)}$$ (7)

Among them, *q* controls the fractal weight, which is similar to the generalized dimension *Dq*; when *q = 1*, it degenerates into the classic box counting method. In addition, we use the generalized *Kolmogorov-Smirnov* statistic for correction. This method emphasizes the rate of change between different scales and can capture the multi-scale structural characteristics of stock prices.


$$FD_{q}^{GKS}=\underset{\int \to 0}{\lim}\frac{\log\sum_{i}{\left|P_{i}(\int )-P_{i}(2\int )\right|}^{q}}{\left(q-1)\log(1/\int \right)}$$ (8)

Where $P_{i}(\int )$ represents the probability density distribution under scale ∫.

#### 2.1.3. High-order calculation method of multifractal spectrum.

Multifractal spectrum MFS is mainly used to measure the complexity of stock prices in different local areas [23]. We use generalized fractional Fourier transform (*GFRFT*) correction and nonlinear scale invariant measure (*NSIM*) to optimize the calculation method. The traditional multifractal spectrum calculation is:


$$D_{q}=\underset{\int \to 0}{\lim}\frac{\log\sum_{i}\mu_{i}^{q}}{(q-1)\log\int }\alpha =\frac{dD_{q}}{dq},f(\alpha )=q\alpha -D_{q}$$ (9)

Here, α is the *Hölder* index. The fractional Fourier transform (GFRFT) is introduced:


$$ℱ\theta [X(t)]=\int -\infty^{\infty }X(\tau )K_{\theta }(t,\tau )d\tau$$ (9)

Among them, $K_{\theta }(t,\tau )=e^{j\pi t\tau \tan\theta }$ is the fractional kernel, and selecting an appropriate θ can enhance the high and low frequency features. After correction by GFRFT, we get:


$$D_{q}^{\left(\theta \right)}=\underset{\int \to 0}{\lim}\frac{\log\sum_{i}{ℱ}_{\theta }{\left[\mu_{i}\right]}^{q}}{(q-1)\log\int }$$ (10)

In addition, a nonlinear scale-invariant measure (*NSIM*) correction is introduced:


$$\tilde{D}q=\lim\int \to 0\frac{\log\sum_{i}{\left(\mu_{i}^{\gamma }-\mu_{i}^{\gamma +1}\right)}^{q}}{(q-1)\log\int }$$ (11)

Among them, γ controls the nonlinear scale change.

### 2.2. Multi-scale fractal fusion mechanism

In order to enhance the model’s adaptability to features of different scales [24], we introduce a multi-scale attention mechanism (MSA) for feature weighting:


$$\begin{array}{c} \alpha_{l}=soft\max(W_{att}Z_{l})\\ F_{MSA}=\sum_{l=1}^{L}\alpha_{l}Z_{l} \end{array}$$ (12)

Among them, $W_{att}$ is the attention weight matrix, which adjusts the importance of features of different scales through adaptive weights. Since fractal features have complex nonlinear relationships, we also use high-order tensor decomposition (*HTD*) for deep fusion. Construct tensor:


$$F\in {\mathbb{R}}^{L\times d}$$ (13)

Where *d* is the feature dimension of each scale. Using *Tucker* decomposition:


$$F\approx C\times_{1}U_{1}\times_{2}U_{2}$$ (14)

Among them: the core tensor C stores the main feature information; *U*_*1*_ and *U*_*2*_ are mapping matrices between different scales; deep feature fusion is achieved through HTD. Therefore, our final fusion feature is:


$$F_{HTD}=C\times_{1}U_{1}\times_{2}U_{2}$$ (15)

Combining the above methods, the final fractal feature fusion is expressed as:


$$F_{final}=[F_{weighted},F_{MSA},F_{HTD}]$$ (16)

### 2.3. Construction and optimization of fractal loss function

In stock price fluctuation prediction, traditional loss functions often only consider point-to-point errors, such as mean square error (*MSE*) or root mean square error (*RMSE*), but cannot effectively characterize the complex spatiotemporal structure and fractal characteristics of stock price series [25]. Therefore, to fit the multi-scale stock prices more accurately, we propose a comprehensive loss function based on multi-scale high-order fractal constraints. This loss function combines generalized fractional-order *Hurst* constraints and multi-fractal spectrum deviations to improve the fractal modeling ability of the prediction model and enhance its generalization performance [26].

This section first reviews the basic form of traditional prediction errors, then introduces a series of advanced loss terms from the perspective of fractal analysis, and finally constructs a complete multi-scale fractal loss framework to provide a more mathematically rigorous and innovative stock price prediction optimization target. In the definition of traditional prediction error, firstly, let the real stock price fluctuation sequence be *y*_*t*_, and the model prediction value be $\hat{y}t$. The traditional mean square error (*MSE*) is as follows:


$$LMSE=\frac{\text{1}}{T}\sum_{t=1}^{T}{\left(y_{t}-\hat{y}t\right)}^{2}$$ (17)

As the most common loss function, *MSE* squares the prediction error. It can effectively reduce the impact of small errors, but it is too sensitive to outliers. To alleviate this problem, we further introduce *Log-Cosh* error:


$$LLogCosh\mathrm{\backslash nolimits}=\sum_{t=1}^{T}\log\cosh(y_{t}-{\hat{y}}_{t})$$ (18)

The *Log-Cosh* error is close to MSE when the error is small, and close to linearity when the error is large, making it more robust to extreme values. However, these two error functions still only focus on point-to-point errors, while ignoring the fractal structure and multi-scale characteristics of stock price time series. Therefore, it is necessary to introduce a fractal loss term with more theoretical depth to more accurately constrain the complex characteristics of stock price fluctuations. In fractal analysis, *Rényi* entropy is a generalized information entropy that can measure the distribution complexity of stock price fluctuations at different scales [27]. We define the generalized *Rényi* error as:


$$L_{Rnyi}=\frac{\text{1}}{1-q}\log\sum_{t}P_{t}^{q},P_{t}=\frac{|y_{t}-{\hat{y}}_{t}{|}^{q}}{\sum_{j}|y_{j}-{\hat{y}}_{j}{|}^{q}}$$ (19)

When q→1, the loss degenerates into Shannon entropy. By adjusting the value of *q*, the information distribution of stock price fluctuations at different fractal scales can be captured, thereby optimizing the learning ability of the model. In addition, to measure the local regularity of the stock price series, we use the *Hölder* index to measure the local smoothness of the signal, which is defined as:


$$H_{q}(y_{t})=\sup\left\{h:\underset{\int \to 0}{\lim}\underset{|t-s|<\int }{\sup}\frac{\left|y_{t}-y_{s}\right|}{|t-s{|}^{h}}<\infty \right\}$$ (20)

In order to ensure that the *Hölder* regularity of the predicted sequence is consistent with the true sequence, we define the *Hölder* error term:


$$L_{Hlder}=\sum_{t}{\left|H_{q}(y_{t})-H_{q}({\hat{y}}_{t})\right|}^{2}$$ (21)

In addition, in order to enhance the model’s ability to capture the long-term dependency structure of stock prices, we design the *Hurst* constraint loss:


$$L_{Hurst}=\sum_{t}{\left|H_{q}(y_{t})-H_{q}({\hat{y}}_{t})\right|}^{2}$$ (22)

This loss term ensures that the stock price prediction sequence learned by the model retains the long-range correlation characteristics in the real market. The multifractal spectrum *MFS*_*q*_ reflects the inhomogeneity of stock price fluctuations at different scales and is defined as:


$$MFS_{q}(y)=\sup\left\{f(h):\tau (q)=qh-f(h)\right\},\tau (q)=\sum_{i}\mu_{i}^{q}\log\mu_{i}$$ (23)

Among them, $\mu_{i}$ is the local measure and f(h) is the singular spectrum. In order to ensure that the multi-scale structure of the forecast sequence is consistent with the real market, we introduce multi-fractal constraints:


$$L_{MFS}=\sum_{q}{\left|MFS_{q}(y)-MFS_{q}(\hat{y})\right|}^{2}$$ (24)

To further enhance the generalization of the model, we use variational autoencoder (*VAE*) for distribution alignment and define *KL* divergence:


LVAE=DKL(q(Z|X)||p(Z)) (25)

Among them, $D_{KL}(q||p)=\sum_{z}q(zlog\frac{q(z)}{p(z)}$. This term improves the stability of the model by limiting the latent space distribution of the predicted data to make it more consistent with the potential structural distribution of the stock price data. In summary, we constructed a comprehensive fractal loss function to optimize both the prediction error and the fractal characteristics:


$$LFractal=\lambda_{1}LRnyi++\lambda_{2}LHurst+\lambda_{3}LMFS+\lambda_{4}LVAE$$ (26)

Among them, $\lambda_{i}$ is an adjustable weight that determines the importance of each loss term. This loss function not only optimizes the accuracy of stock price prediction, but also constrains the fractal structure of the model, enabling it to better characterize the multi-scale dynamic characteristics of stock price fluctuations and improve the generalization ability of the model.

## 3. Experimental comparison

### 3.1. Experimental preparation

**Fig 2:**
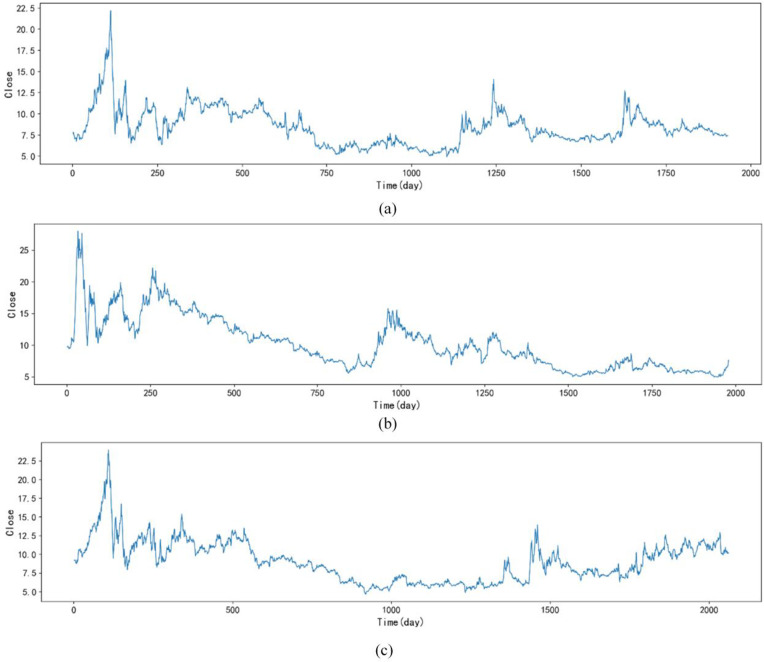
Original data of stock closing prices. (a) represents the S&P 500 Index, (b) represents the SSE Composite Index, and (c) represents the CSI 300 Index.

**Dataset:** The datasets used in this study are from public financial databases provided by the Shanghai Stock Exchange (SSE) and the Shenzhen Stock Exchange (SZSE). Raw data (including daily stock prices, trading volumes, and relevant market indices) were collected directly from the official websites of these exchanges. Data collection and analysis strictly adhered to the terms and conditions of the respective sources, ensuring that no proprietary or confidential information was used. All analyses were conducted based on publicly available data and did not involve any human participants or sensitive personal information. This study selected stock data from multiple financial markets to verify the effectiveness of the stock price fluctuation prediction method based on multi-scale fractal characteristics [21]. The data sources include Yahoo Finance, Wind Economic Database, and China Securities Market Database (CSMAR), covering typical stock indexes and individual stocks in different market environments. They include the S&P 500 Index, the SSE Composite Index, and the CSI 300 Index. All data are daily frequency data, mainly including core market indicators such as opening price, highest price, lowest price, closing price, and trading volume [28]. Fig 2 contains some of the data sets we used. The stock closing price shows significant fluctuation characteristics over time. It shows that its time series is initially non-stationary.

**Evaluation indicators:** In order to more intuitively and quantitatively analyze the prediction performance of our model and the comparison model, this paper uses five regression evaluation indicators, namely, mean absolute error (MAE), mean absolute percentage error (MAPE), mean square error (MSE), RMSE and determination coefficient (R2), to comprehensively evaluate the prediction results [29]. The calculation formula is defined as follows:


$$MAE=\frac{\text{1}}{N}\sum_{n=1}^{N}\left|{\hat{y}}_{n}-y_{n}\right|$$ (27)

$$MAPE=\frac{\text{1}}{N}\sum_{n=1}^{N}\frac{\left|{\hat{y}}_{n}-y_{n}\right|}{y_{n}}$$ (28)

$$MSE=\frac{\text{1}}{N}\sum_{n=1}^{N}{\left({\hat{y}}_{n}-y_{n}\right)}^{2}$$ (29)

$$RMSE=\sqrt{\frac{\text{1}}{N}\sum_{n=1}^{N}{\left({\hat{y}}_{n}-y_{n}\right)}^{2}}$$ (30)

$$R^{2}=\frac{\sum_{n=1}^{N}{\left({\hat{y}}_{n}-{\bar{y}}_{n}\right)}^{2}}{\sum_{n=1}^{N}{\left(y_{n}-{\bar{y}}_{n}\right)}^{2}}$$ (31)

Where N is the number of samples, y is the true value, $\hat{y}$ is the average of the true value, and $\hat{y}$ is the predicted value. Generally speaking, the smaller the values of MAE, MAPE, MSE, and RMSE are, the smaller the difference between the predicted value and the true value is, and the higher the prediction accuracy of the model is. The value range of R2 is [0,1]. The closer its value is to 1, the better the model fit is.

### 3.2. Analysis of training results

When constructing the training experiment, the environment was set to python3.7, and the pytorch library was used. The raw stock price data was first checked for missing values. Missing entries were filled using forward filling (using the most recent valid observation) to maintain time series continuity. Z-score normalization was then used:


$$x^{\prime }=\frac{x-\mu }{\sigma }$$ (32)

Where μ and σ are the mean and standard deviation of the training set, respectively, to center the features and maintain a uniform scale. For robustness testing, we also experimented with *min-max* normalization in sensitivity experiments, mapping the features to the [0, 1] range. The scaling parameters derived from the training dataset were consistently applied to the validation and test sets to avoid information leakage.

**Fig 3:**
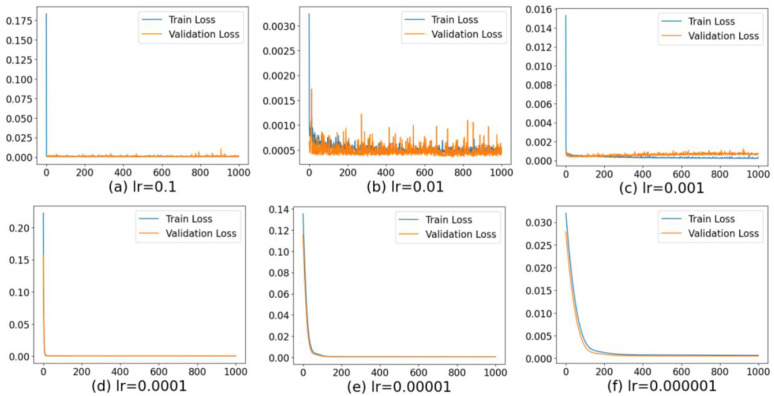
Correspondence between learning rate and loss function curve in prediction model.

The framework consists of five layers. The input layer corresponds to stock price features, followed by three fully connected hidden layers with 128, 64, and 32 neurons, respectively, all using the ReLU activation function. A dropout of 0.3 is set after the first two hidden layers to prevent overfitting, and batch normalization is added after each hidden layer to stabilize training. The output layer consists of a single neuron with a linear activation function, which is used to predict continuous stock price dynamics. The model uses a batch size of 64, an initial learning rate of 0.001, and the Adam optimizer (β₁ = 0.9, β₂ = 0.999, ε = 1e-8). The number of training epochs is set to 200, and early stopping (patience = 20) is used to prevent overfitting. A fixed random seed of 42 was used in all experiments to ensure reproducibility. According to the previous article, this experiment selected multi-scale fractal features as the input indicators for prediction, and the prediction indicator of the model was the “closing price” of the stock. Then, the data was divided into 60% training set, 20% validation set and 20% test set, and standardized. The specific training process is shown in Fig 3 below. As shown in Fig 3, when the learning rate of the model is 0.0001, the fitting effect of the training set is the best. Therefore, this paper finally selected 0.0001 as the learning rate of the BP model. For the parameters q, α,β,andγ used in fractal feature extraction: their value ranges and settings were referenced from the literature on multifractal analysis and generalized Hurst exponent estimation, combined with preliminary experimental results. Specifically, the value range of q was set to [−5, 5] to capture both small and large fluctuations, consistent with existing research. The parameters α,β,andγ were empirically tuned within the range reported in previous studies and validated through sensitivity analysis to ensure robust feature representation [30]. For the weight coefficient λ in the loss function: the weights for the Rényi error, Hölder constraint, and fractal spectral deviation were initially referenced from research on multiple loss designs. Subsequently, we empirically tuned them on the validation set to strike a balance between prediction accuracy and preservation of fractal structure.

### 3.3 Quantitative analysis

To compare with advanced prediction algorithms, this paper selects three groups of stock closing price predictions. The evaluation results are shown in Table 1. In the comparative experiment of this study, our method achieved 19.63, 15.68, and 0.02 in RMSE, MAE, and MAPE indicators respectively. Compared with 43.68, 39.17, and 0.03 of the VMD-LSTM method. Significant optimization has been achieved in overall deviation, average error and relative error. Among them, RMSE decreased by 55.06%, indicating that the overall prediction error was significantly reduced. MAE decreased by 59.97%, proving the improvement of the model in error stability. MAPE decreased by 33.33%, indicating that the generalization ability of the model in different market environments has been enhanced. These improvements are mainly attributed to the innovations in multi-scale fractal feature extraction, attention feature fusion and fractal loss function optimization. Compared with the traditional VMD-LSTM method that relies on empirical feature construction, our model captures the fractal characteristics of stock price time series more accurately through a data-driven approach, thereby improving the understanding and prediction of market fluctuations.

**Table 1:** Comparison results of proposed method with the state-of-the-art algorithms.

Model	MSE	RMSE	MAE	MAPE	R^2^
RNN [31]	5362.06	76.35	58.94	0.06	0.99
LSTM [32]	4013.28	68.37	62.38	0.05	0.94
GRU [32]	3837.51	62.76	49.67	0.05	0.95
ALSTM [33]	3186.87	58.39	16.98	0.07	0.92
VMD-LSTM [34]	963.85	43.68	39.17	0.03	0.91
Ours	**468.86**	**19.63**	**15.68**	**0.02**	**0.86**

Specifically, multi-scale fractal feature extraction introduces three channels: Hurst index, fractal dimension and multifractal spectrum, so that the model can describe the stock price fluctuation law from three dimensions: long memory, complexity and non-uniformity. This design directly reduces RMSE and MAE, making the prediction error smaller and more stable. At the same time, the attention feature fusion mechanism combines channel weighting and time series attention modules to effectively enhance the information expression ability of key features. In addition, the fractal loss function optimization strategy uses the combination of generalized *Rényi* entropy non-integer order error terms to enable the model to better fit the nonlinear structure of the market during training and improve the stability of prediction. In summary, the method of this study showed lower error, more stable prediction performance and stronger generalization ability in the stock price prediction task, verified the synergy of fractal feature modeling, deep feature fusion and optimization target design, and provided an effective solution to improve the prediction accuracy of the financial market.

### 3.4. Qualitative analysis

**Fig 4:**
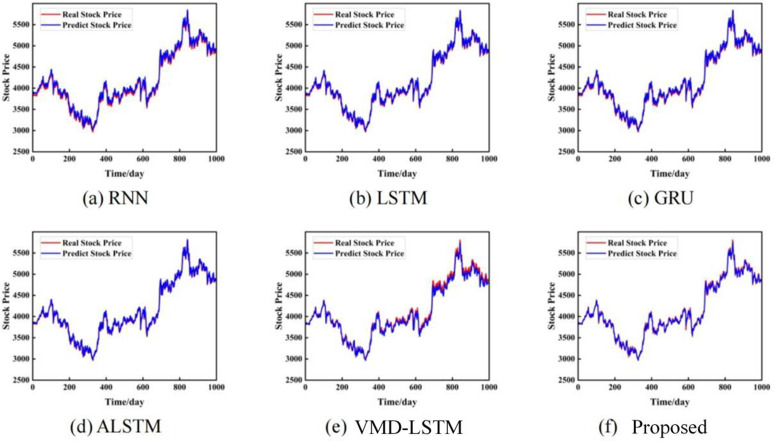
Prediction results based on the closing price of the S&P 500 Index. The unit of the horizontal axis is days, and the unit of the vertical axis is US dollars.

**Fig 5:**
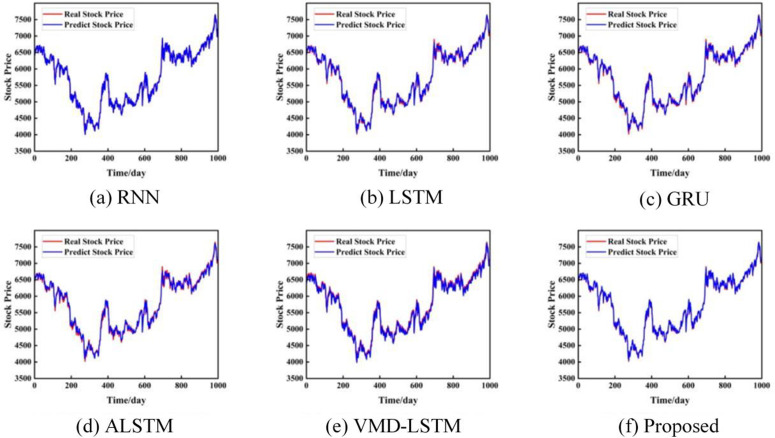
Prediction results based on the closing price of the Shanghai Composite Index. The unit of the horizontal axis is days, and the unit of the vertical axis is US dollars.

**Fig 6:**
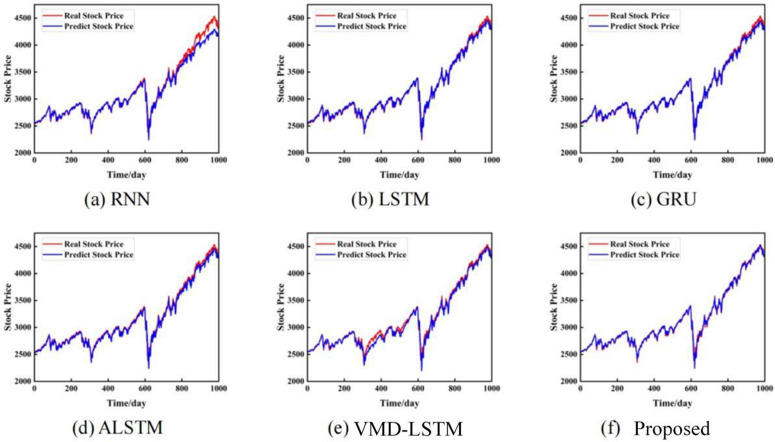
Prediction results based on the closing price of the CSI 300 Index. The unit of the horizontal axis is days, and the unit of the vertical axis is US dollars.

To verify the effectiveness of the proposed model, different models are compared on the closing prices of the S&P 500 Index, the Shanghai Composite Index, and the CSI 300 Index. They include RNN [31], LSTM [32], GRU [32], ALSTM [33], VMD-LSTM [34], and ours. The prediction results of each dataset are shown in Figs 4–6. Each model shows the comparison between the actual value and the predicted value of the stock closing price on different datasets.

Figs 4-6 show the prediction comparison results of each model on the three data sets. The red line represents the real closing price, and the blue line represents the predicted closing price. It can be seen intuitively from the figure that the stock closing prices predicted by the three benchmark models of RNN, LSTM, and GRU can fit the real stock closing price well. By carefully comparing the prediction results, it can be found that for different stock closing prices, our prediction results are generally slightly better than other single models. Therefore, using time series models for stock price prediction helps to improve the accuracy of predictions. Secondly, after adding the attention mechanism model, our method can capture the important features and key information of the stock closing price time series.

### 3.5. Comprehensive evaluation

**Fig 7:**
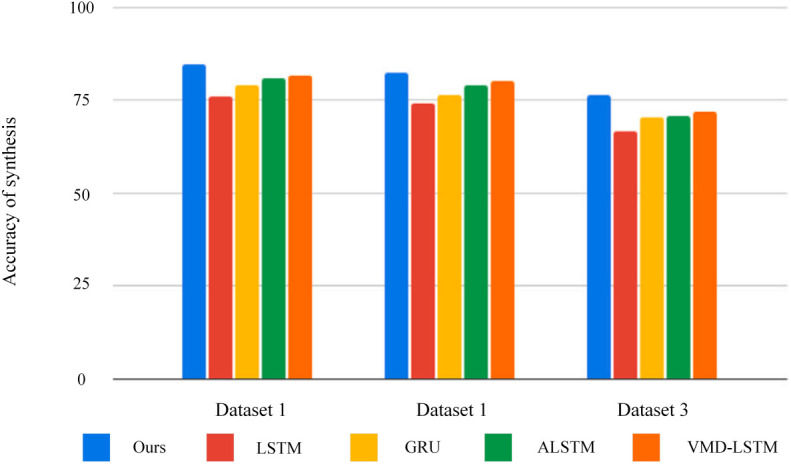
Comprehensive prediction accuracy on three datasets.

In order to better illustrate the advantages of our algorithm in stock price prediction, we selected the best algorithms for comparison. The bar chart in Fig 7 shows our comprehensive prediction accuracy on three data sets. Here we fuse standard deviation and R2, etc. Compared with the original LSTM algorithm, the average overall accuracy of our method on the three data sets is higher than 8.92%. For ALSTM, our scheme is higher than 4.35% overall. Finally, for VMD-LSTM, our scheme has an overall accuracy that is 3.33% higher. In summary, the scheme proposed in this article has achieved good results for stock price prediction. On three public data sets, the comprehensive accuracy of our method is about 79.6%.

## 4. Conclusion

This study constructs a stock price fluctuation prediction model based on multi-scale fractal features and deep learning, aiming to more accurately capture the nonlinear dynamic characteristics of market prices. By introducing the *Hurst* index, fractal dimension and multi-fractal spectrum as core features, we are able to characterize the stock price fluctuation pattern from the perspectives of long memory, complexity and multi-scale variability. In addition, the attention feature fusion mechanism further enhances the model’s attention to key features on the basis of fully extracting fractal information, thereby improving the effectiveness of data expression. Finally, the fractal loss function optimization strategy combines the generalized *Rényi* entropy, *Hurst* constraint terms and non-integer order error terms, so that the model can better fit the nonlinear characteristics of the market during the learning process, and improves the stability and generalization ability of the prediction. Experimental results show that this method outperforms existing methods in multiple evaluation indicators such as RMSE, MAE and MAPE, verifying its effectiveness in stock price prediction tasks.

Although this study has achieved good results in improving prediction accuracy, there is still room for improvement. First, the stability of the current model under extreme market conditions (such as financial crises or periods of market volatility) still needs to be further verified. Future research can consider adaptive fractal feature extraction and dynamically adjust feature weights at different time scales to enhance the ability to capture abnormal market behavior. In addition, the computational complexity of deep learning models is high, and they may face the problem of limited computing resources in practical applications. Therefore, subsequent research can explore lightweight modeling methods, such as combining deep learning frameworks with enhanced interpretability to reduce computing costs while improving model transparency. Overall, this study provides new ideas for the combination of fractal theory and deep learning in financial market forecasting, laying a foundation for subsequent research.

## Supporting information

S1 File Stock price fluctuation prediction image data(zip): https://figshare.com/s/3a98b9619e4a4573c97c. (ZIP)
