# Supplementary figures and images for: Stock price dynamics prediction based on multi-scale fractals and deep learning
Source: PLoS One. 2025 Dec 2;20(12):e0335554. doi: 10.1371/journal.pone.0335554 (PMC12671762; doi:10.1371/journal.pone.0335554)

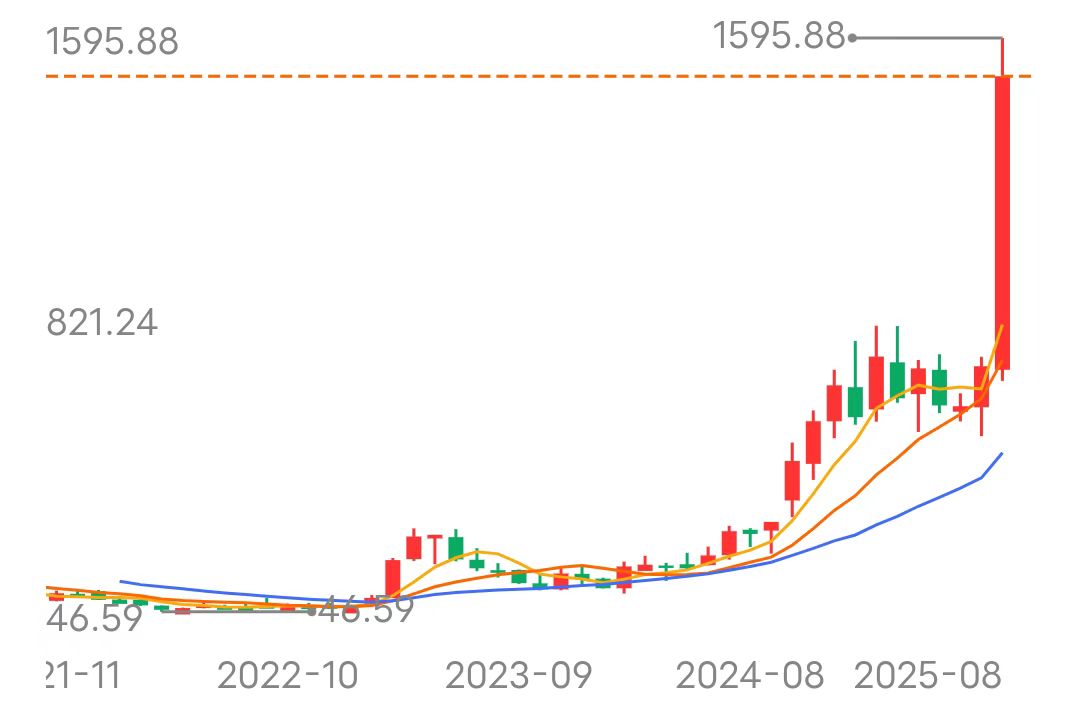

Supplement: S1 File — (ZIP) [file pone.0335554.s001.zip › supporting information/stocks figure/17f1cbd16ec13fda2f4a26dd7aa6183d.jpg]

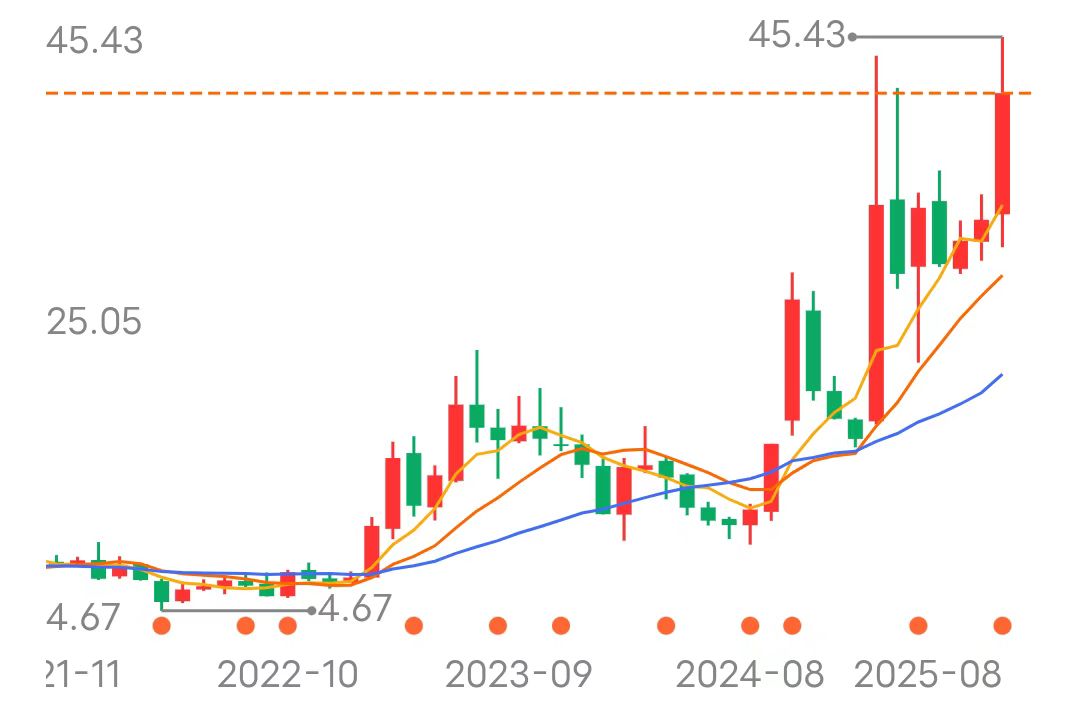

Supplement: S1 File — (ZIP) [file pone.0335554.s001.zip › supporting information/stocks figure/1ce5abf16e69a69d5ca72e6dbecbffc9.jpg]

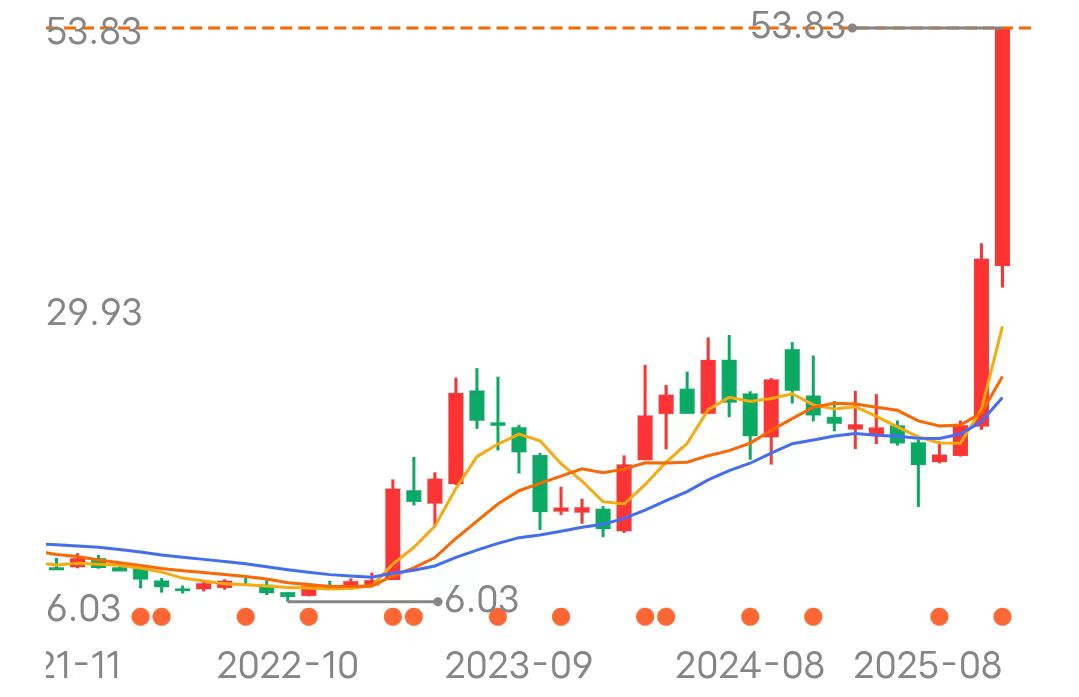

Supplement: S1 File — (ZIP) [file pone.0335554.s001.zip › supporting information/stocks figure/4a44d0cc5b5570e221132cd01413a9de.jpg]

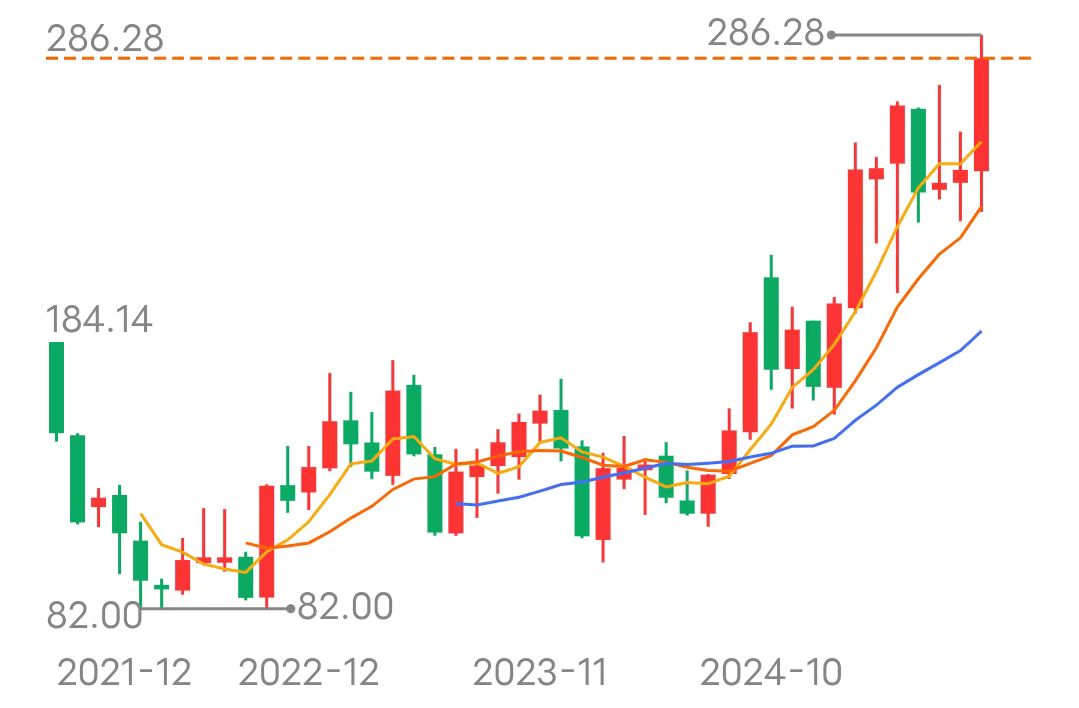

Supplement: S1 File — (ZIP) [file pone.0335554.s001.zip › supporting information/stocks figure/7242190b6faa4662f0a647a5a3a4bd69.jpg]

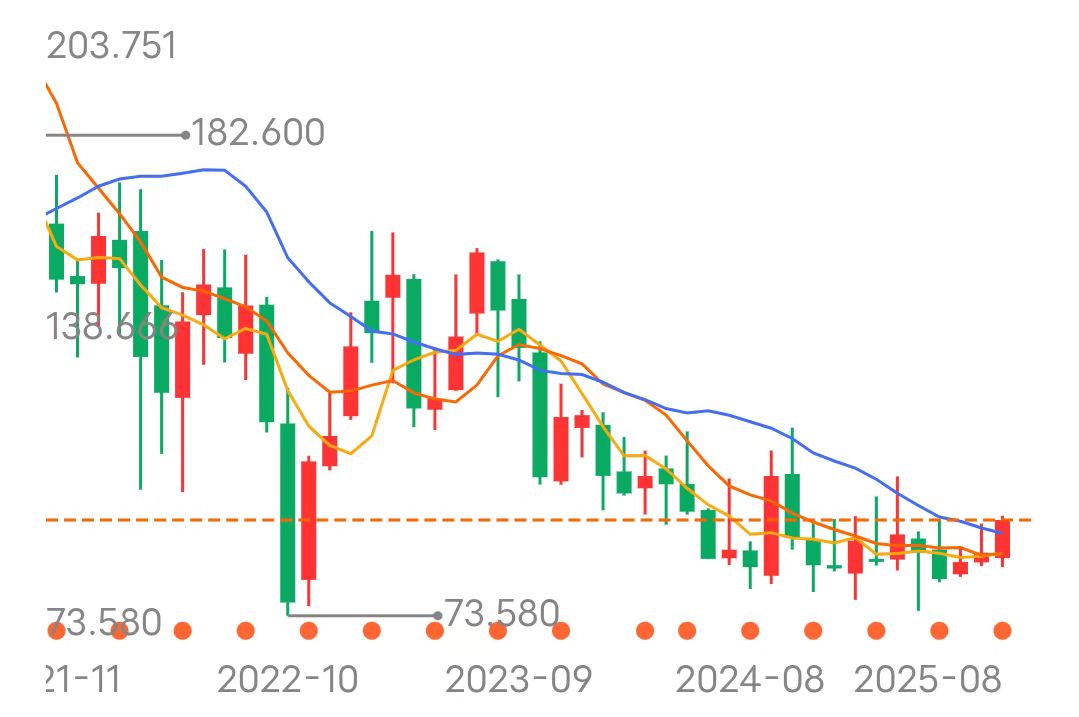

Supplement: S1 File — (ZIP) [file pone.0335554.s001.zip › supporting information/stocks figure/ae0e01fd2e687dc5a1ba66b8f164ca1e.jpg]

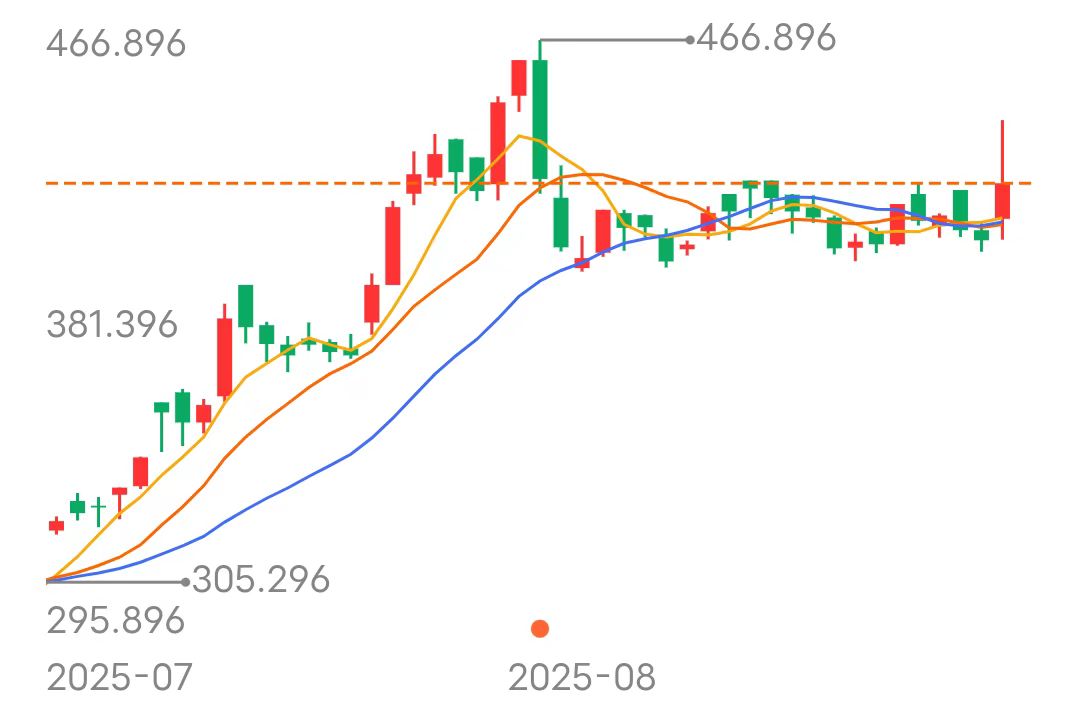

Supplement: S1 File — (ZIP) [file pone.0335554.s001.zip › supporting information/stocks figure/b35d4b8dbdcb78574ee8c4497ac8a6de.jpg]

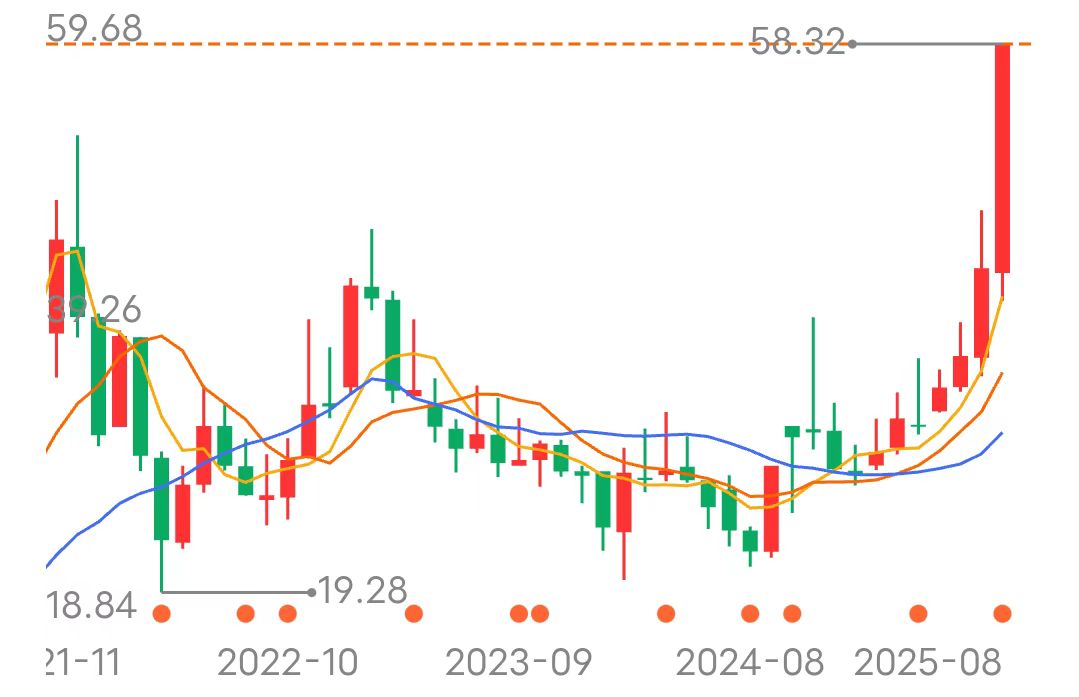

Supplement: S1 File — (ZIP) [file pone.0335554.s001.zip › supporting information/stocks figure/b8e400502c46b5c155c4dec2671e3004.jpg]

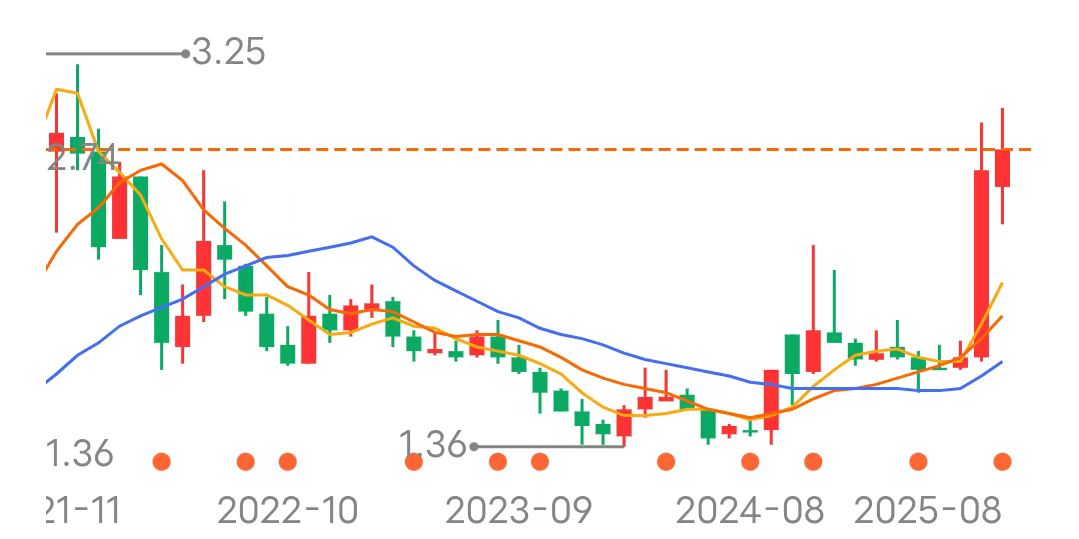

Supplement: S1 File — (ZIP) [file pone.0335554.s001.zip › supporting information/stocks figure/f2107c37acfa926d309d8e353d4f665e.jpg]

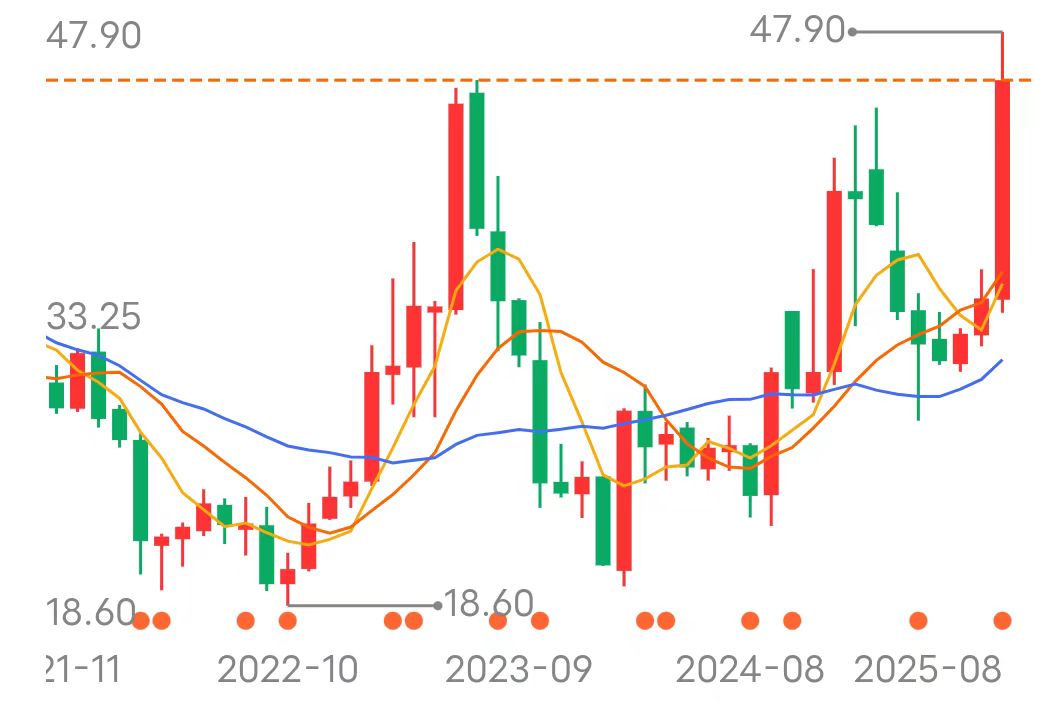

Supplement: S1 File — (ZIP) [file pone.0335554.s001.zip › supporting information/stocks figure/ffcfbdf419c190558588c74e5e9b282c.jpg]
